# Effect of wither application of an analogue of pig appeasing pheromone on encounters between unfamiliar mini-pigs

**DOI:** 10.1186/s40813-022-00294-7

**Published:** 2022-12-14

**Authors:** Míriam Marcet-Rius, Tiago Mendonça, Patrick Pageat, Sana Arroub, Cécile Bienboire-Frosini, Camille Chabaud, Eva Teruel, Alessandro Cozzi

**Affiliations:** IRSEA (Research Institute in Semiochemistry and Applied Ethology), Quartier Salignan, 84400 Apt, France

**Keywords:** Agonistic behaviour, Fighting in pigs, Mixing of pigs, Appeasing pheromones, Prosocial behaviours, Animal welfare, Salivary alpha-amylase, Salivary cortisol

## Abstract

**Background:**

The practice of mixing unfamiliar pigs on farms is common but results in fighting, welfare problems and performance issues. Pigs have different ways of resolving social conflicts, including aggressive and affiliative behaviours. Synthetic appeasing pheromones have demonstrated many positive effects in animal husbandry and are regularly used by breeders to improve animal welfare and performance. The aim of the study was to investigate the effect of a new method of applying pig appeasing-pheromone (PAP) to the withers in an experimental model of pig mixing to determine whether PAP reduced aggression and fighting, increased prosocial behaviours, and improved behavioural and physiological indicators of welfare.

**Results:**

PAP reduced fighting between mini-pigs (df = 1; F = 13.47; *P* = 0.001; mixed logistic regression). Even if not significant, agonistic behaviours tended to be reduced when the treatment was applied (df = 1; F = 4.14; *P* = 0.058; mixed logistic regression). Likewise, mini-pigs seemed to be scored as not aggressive at all (df = 1; F = 3.61; *P* = 0.070; GLMM) and to be less aggressive toward the other pig than when placebo was applied. Concerning the latency of the first contact without aggression, a significant effect was found between the PAP and placebo groups (df = 1; *χ*^2^ = 4.74; *P* = 0.0295; Cox model). Moreover, even if not significant, the treated mini-pigs seemed to spent more time looking at each other (df = 1; F = 3.59; *P* = 0.071; GLMM) and immobile and/or ground sniffing (df = 1; F = 3.18; *P* = 0.088; GLMM) than those that received placebo. No significant difference was found between groups for salivary cortisol concentration (df = 1; F = 0.10; *P* = 0.752; GLMM), but variances between groups were heterogeneous at every time. No significant difference was found between groups regarding alpha-amylase activity (df = 1; F = 0.25; *P* = 0.621; GLMM), but variances between groups were heterogeneous at T0, T1 and T3. These results indicate that the variability (dispersion) within each group was lower when PAP was applied than when the placebo was applied.

**Conclusions:**

The new method of applying PAP improved welfare of mini-pigs (as models of domestic pigs) by reducing fighting, among other interesting results. PAP seems thus a promising biomimetic tool to enhance animal welfare in pig production systems.

## Background

Mixing unfamiliar pigs, which is routinely done in pig farms, is known to greatly increase fighting as well as decrease pig growth and performance [1, 2]. Thus, there is a need to find useful and feasible solutions in this system to reduce fighting, which would directly improve animal welfare and production [3].

Gregarious animals that live in permanent social groups experience intra-group competition. Conflicts over resources may result in highly aggressive behaviours, and in some conditions, non-dispersive forms of conflict resolution can appear [4]. When unfamiliar animals meet, social conflict usually arises, especially in form of aggression. Aggression in pigs, and more precisely, agonistic behaviour, consists of actively displacing another pig, ramming it, or pushing it with the head, aggressively biting any part of the other pig or actively pursuing it [5, 6]. Agonistic behaviour is nevertheless essential for the establishment of a dominance hierarchy among new group members [7], to ensure the well-functioning of the group in the nature. Fighting between two pigs consists of attacks including head-to-head blows and head-to-body blows, shoving, biting, and/or physical displacements by the aggressor pig [8]. Aside from aggression, agonistic behaviours and fighting, other behavioural mechanisms may resolve social conflicts [9]. For example, affiliative behaviours promote reconciliation and group cohesion [4, 9, 10]. Reconciliation mechanisms have been studied in many different species, such as primates [11], dolphins [12], goats [13], hyenas [14], dogs [15], wolves [16], rooks [17] and horses [4]. Nevertheless, they remain understudied in other species, such as pigs [9].

Social interactions are essential for the above welfare of animals, including both the mental and the physical health [18]. Linked to social interactions, there is a family of behaviours, still poorly known in many species, named as prosocial behaviours. They are defined as actions that an individual performs to benefit others [19]. They could be considered as positive welfare indicators of a bilateral nature, as they are beneficial to the recipient but also rewarding to the donor by letting him/her feel positive emotions [20]. The prosocial behavioural repertoire includes affiliation (e.g. preferential interactions, spatial proximity and behavioural synchronisation [21]), cooperation, parental care, sharing, teaching, and other types of caring and helping behaviours towards others among others [18]. Some concrete examples of prosocial behaviours in pigs are social nosing between them in particular contexts [22] and spatial proximity [9].

Several physiological measures exist to assess animal welfare and stress. Stress-related reactions have at least two principal components: the first one involves corticotropin-releasing hormone, activating the limbic–hypothalamic‒pituitary‒adrenal (LHPA) axis, and the secretion of glucocorticoids into circulation; the second one involves the activation of the autonomic (sympathetic) nervous system and the release of catecholamines into the bloodstream [23, 24]. Those physiological measures linked to stress response need to be combined with other parameters, such as the behavioural ones, to be able to understand the context and interpret the results. In pigs, cortisol is the major glucocorticoid produced in the adrenal cortex [25]. Its production has a circadian rhythm [26, 27], but it rises independently of it in response to stress [28], as well as in response to other factors, such as sexual activity, the anticipation of feeding or excitement. Studies consistently report high correlations between serum and salivary cortisol, indicating that salivary cortisol levels reliably estimate serum cortisol levels [29–31]. Concerning the other component of the stress response, alpha-amylase has been identified as a biomarker that appears to indicate stress. Secretion of alpha-amylase from the salivary glands is controlled by autonomic nervous signals, and studies have shown that levels of salivary alpha-amylase increase under a variety of physically and psychologically stressful conditions in human subjects [32] and in pigs [33]. Interestingly, studies have shown that cortisol concentration often does not correlate with alpha-amylase activity during stress [34–36].

Appeasing pheromones were initially discovered in pigs and have been shown to reduce agonistic behaviour in piglets [37], weaners [38, 39], and sows [40], among many other positive effects [e.g., 41], such as the inhibition of cortisol augmentation during social stress in adult mini-pigs [42]. Some examples of the practical application of maternal (appeasing) pheromones are a decrease in agonistic behaviour and stimulation of feeding behaviour that results in greater weight gain in piglets [43]. A synthetic analogue of pig appeasing pheromone is available on the market and is commonly used by farmers in the pig industry [44]. The application of appeasing pheromones as pheromonotherapy simplifies treatment for anxiety and phobia-related issues in various species, such as dogs, cats [45–47], rabbits [43, 48], and horses [49, 50], reduces aggression among cats [51] and among dogs [52], and increases the welfare and performance of dairy cattle [53, 54], beef cattle [55–57], and chicks [58], among others.

The aim of the present study was to investigate the effect of applying the new form of pig appeasing pheromone (PAP) (SecurePig Flash®, SIGNS Labs, France) to the skin of the withers in a model of pig mixing based on mini-pigs, a model of domestic commercial pig [59]. This new application of the PAP (administered individually on the withers skin instead of in block diffusers that are placed throughout the rooms) seems to produce an almost immediate effect (a “flash” effect) compared to the classic diffusion in blocks, as it is the same individual who diffuses it once it is applied, meaning that it could have an appeasing effect during social interactions. This area is easily reached by people applying the treatment but not for the animals. Thus, they cannot touch it or lick it. Numerous farmers in the pig industry have already expressed their contentment with the product, but to our knowledge, this is the first scientific study about it. The precise aims of the study were to determine whether (i) it reduces aggression and fighting, (ii) it increases prosocial behaviours, and (iii) it impacts behavioural and physiological indicators of animal welfare.

## Methods

### Animals and housing

The mini-pigs (*Sus scrofa domesticus*) (*n* = 12: castrated males = 5, females = 7; age = 4 years) involved in the study were originated from cross-breeding Asian miniature breeds (Vietnamese and Chinese) with conventional pig white-hair breeds (Landrace and Large White). These pigs came from Specipig (a centre for breeding and biomedical research) in Barcelona, Spain. Animal keepers, technicians, and veterinarians provided care for the pigs. They were housed in two identical rooms (30 m^2^) with controlled environmental parameters: a mean temperature of 22 °C, artificial ventilation, and 50–60% humidity. In each room, the pigs were housed in pairs in 1.85 × 1.35 m pens (2.5 m^2^). Thus, in each room (group), there were three pens with two minipigs (so a total of six minipigs per group) participating in the study. They were fed twice a day with a special diet for mini-pigs (Special Diets Services, Paris, France) and had continuous access to water. Lighting was provided from 7.00 a.m. to 6.00 p.m.

### Treatment application

On Monday of the first week of the trial, at 3 p.m., 5 ml of treatment A or B was applied individually on the withers skin of all the mini-pigs of the first group, according to the manufacturer’s instructions (SIGNS Labs, France). It was a blinded procedure; thus, the operator did not know if the treatment was either the PAP (SecurePig Flash®, SIGNS Labs, France) or placebo. The placebo is a product that has exactly the same shape, odor, color, and is contained in a comparable bottle. Practically speaking, the "placebo" is just the vehicle proposed in the same container as the tested product. It prevents the observers to be possibly influenced by knowing what treatment is applied. Then, the treatment (A or B) was applied every Monday at the same time, as the persistence of the product on the skin was five days.

### Description of the experimental room

**Fig. 1:**
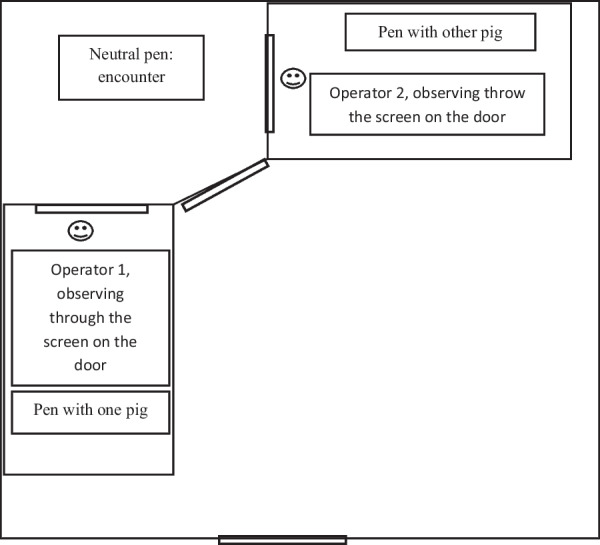
Experimental room. Dimensions: room = 30 m^2^ (6 m × 5 m); neutral pen = 5 m^2^ (2.5 m × 2 m); pens = 4.5 m^2^ (3 m × 1.5 m)

The experimental room measured 30 m^2^ (6 m × 5 m) and was situated just in front of the housing rooms, separated by a corridor that the mini-pigs needed to cross to arrive there (10 m). Inside the room, there were two pens of 4.5 m^2^ (3 m × 1.5 m), one on the left of the entrance and the other on the front, both of them touching the wall (Fig. 1). The neutral pen was situated between these two pens and measured 5 m^2^ (2.5 m × 2 m). The pens and the neutral pen were separated by two doors so that the mini-pigs could go from the pen to the neutral pen when the operator opened it. On those doors, a screen was installed to prevent the pigs from seeing between them and seeing the neutral pen, as well as from seeing the operator. The operator stayed hidden in the pen while the mini-pigs were in the neutral pen so that the pigs could not see them, but they could see the pigs thanks to a small window through the screen. Enough litter (shaving) to cover all the floor was present inside the three pens (two pens and a neutral pen) and refreshed between tests. A drinker was available in each pen but not in the neutral pen. A camera (Sony HDR-CX625) was installed on the ceiling of the neutral pen.

### Experimental protocol

The study took place over five weeks with two parallel groups of comparable mini-pigs in identical conditions: the first group participated in the study for two weeks, then there was one week of pause, and then the second group participated for the following two weeks. Both groups were housed in two identical rooms with exactly the same management during all their lives. In each room, there were six mini-pigs that participated in the study: three castrated males and three females in one room and two castrated males and four females in the other room.

Two different encounters with two unfamiliar pigs from the same group (but different pens) were performed on Tuesday, Wednesday and Thursday. On Friday, the pigs were weighed and received their regular weekly care (cleaning of eyes and ears, deep cleaning of the rooms). The following week, the procedure was the same.

The protocol was as follows: mini-pigs were fed at 8 a.m., and they usually took ten minutes to finish eating. At 9 a.m., saliva was sampled (T_0_). Then, pig number 1 was moved from the housing room to the experimental room: one operator called it to guide it from one room to another, separated by 10 m, and the other operator walked behind it to help guide it; the mini-pigs had been successfully habituated to this procedure during a one-month period by walking them from the housing room to the experimental room. Once pig 1 was installed in pen 1 of the experimental room, pig 2 was moved in the same way, from its pen in the housing room to pen 2 in the experimental room. Moving one pig from one pen to the other took approximately three minutes. When pigs were installed in their pens, they were left there to acclimatise for five minutes. The operators left the room and prepared the material. Then, they came back and started the evaluation of skin lesions.

After that, the encounter was performed: each operator entered each pen, opened the doors at the same time, guided the pigs inside the neutral pen at the same time, and entered again quickly into their pen and hid behind the screen so as not to interfere with the behaviour of the pigs. Nevertheless, they were ready to enter the neutral pen quickly if a fight started to protect the pigs by separating them with a board if necessary. The encounter lasted a maximum of 300 s, but it was stopped by the operators if the pigs started fighting or if one pig attacked (biting or pushing aggressively) the other three times. A total of 24 encounters between unfamiliar pigs were performed, twelve per group. The encounters were recorded by a video camera installed on the ceiling. At the end of the encounter, the two operators entered the neutral pen, and each one guided his or her pig calmly into its pen.

Saliva was sampled (T_1_) just after the end of the encounter with the same procedure, followed by the evaluation of skin lesions following the same protocol to see if new lesions were produced due to the encounter. Afterwards, saliva was sampled again, fifteen minutes after the end of the encounter (T_2_). Elimination (faeces and/or urine) was noted before and after the encounter to evaluate the potential stress of the animals. Finally, the last saliva sampling was performed (T_3_), fifteen minutes after T_2_ (meaning 30 min after the end of the encounter), before moving the mini-pigs to their housing pens following the same described procedure (Table 1).

**Table 1:** Daily schedule with sampling times

Time	Action
3:00–3:15 p.m. (previous day)	Application of treatment to all the mini-pigs
9:00–9:05 a.m	Collection of saliva from the first two mini-pigs (T_0_)
9:05–9:15 a.m	Bringing of the two mini-pigs to the experimental room
9:15–9:20 a.m	5 min of calm for the pigs to acclimatise
9:20–9:30 a.m	Evaluation of skin lesions (before encounter)
9:30–9:45 a.m	Preparation of encounter, encounter, and end of encounter
9:45–9:50 a.m	Collection of saliva (T_1_)
9:50–9:55 a.m	Evaluation of skin lesions (after encounter)
10:00–10:05 a.m	Collection of saliva (T_2_)
10:15–10:20 a.m	Collection of saliva (T_3_)
10:20–10:30 a.m	Bringing of the mini-pigs from the experimental room to the housing room
10:30–11:00 a.m	Cleaning procedure
11 a.m.-1:00 p.m	Re-start of the entire procedure with two other mini-pigs, from the beginning until the end

### Data collection

#### Saliva sampling

Operator 1 sampled the saliva of the pig in pen 1, while operator 2 sampled the saliva of the pig in pen 2 for one and a half minute, using a Salivette® swab (Sarstedt, Numbrecht, Germany) fixed to a clamp, without stressing, touching, or talking to the pig, to avoid any possible influence of the handling. Saliva was sampled to assess cortisol concentration and alpha-amylase activity. As soon as the sample was collected, it was placed in an ice box to keep it at approximately 4 ℃ until it was centrifuged (3 min, 1000 rcf) and then frozen at -20 °C prior to analysis.

The timing of saliva samples was performed according to the literature on alpha-amylase activity and cortisol concentration in pig saliva [33], also considering the kinetics of cortisol passive diffusion from blood to saliva (~ 15–30 min) described in other farm animals [60, 61] (Table 1). The protocol described was performed at the same time every day with the same time intervals for each step.

#### Skin lesions

Skin lesions were evaluated with an adaptation of the Welfare Quality® protocol for pigs [62], as described by Fàbrega et al. [63], each operator with his or her pig. To perform the evaluation, each operator entered the pen and observed the mini-pig, without touching it or talking, and filled the sheet to note the number of lesions already present in each body region before the encounter. The same procedure was performed after the encounter. This procedure lasted approximately two minutes. Prior to the study, the operators had trained together to perform the procedure in a reproducible manner.

#### Behavioural sampling

All the behaviours (walking, immobile and/or ground sniffing, reciprocal look, agonistic behaviour, agonistic signal-threat, fighting, fighting starter, encounter duration, need to stop de encounter, total social nosing, positive and negative social nosing, proximity, tail movement and latency of first contact with and without aggression) were analysed from the video recording according to an ethogram (Table 2) by two independent observers at the end of the study using BORIS software [68].

**Table 2:** Description of the behaviours observed in the video analysis of the encounters by two independent observers

Behaviour	Definition
Walking (duration in s)	Moving its legs in order to advance in any direction of the pen
Immobile and/or ground sniffing (duration in s)	Pig is upright on all four legs, neither moving forwards nor backwards [63], either exploring with the snout and mouth or not
Reciprocal look (duration in s)	Both pigs look at each other at the same moment. The head and the eyes were directed to the other pig. May be a sort of synchronised behaviour, which may indicate a positive welfare state [64, 65]
Agonistic behaviour (binary)	Actively displacing another pig, pushing it, biting it, or actively pursuing it [5, 6]
Agonistic signal–threat (frequency)	Movement of the head or body oriented to the receiver, without contact [66]
Fighting (binary)	Attacks consisting of head-to-head blows, head-to-body blows, shoving, biting and/or physical displacements [8]
Fighting starter (binary)	Pig that starts the fighting by attacking the other pig (head-to-head blows, head-to-body blows, shoving, biting and/or physical displacements) [8]
Encounter duration (in s)	Total duration of the encounter
Need to stop the encounter (binary)	The encounter was stopped when pigs started fighting or when one pig performed three bites on the other. If none of this occurs, the encounter ended at 5 min
Total social nosing (binary)	The pig touches the nose, head or other part of the body of another pig [22]
Positive social nosing (binary)	Social nosing that does not prime aggression after five seconds
Negative social nosing (binary)	Social nosing that primes aggression after five seconds
Proximity (duration in s)	Spatial proximity, being close to each other during specific activities or resting periods. Depending on the context, it could be considered a type of affiliative behaviour [18]. Nine squares divided the test area (3 × 3): when most of the body of both pigs (or all of the body) were in two consecutive squares, thus not separated by another square, we considered that they were in proximity
Tail movement (duration in s)	Lateral tail wagging, mostly from side to side, linked to positive emotions [67]. Each time that the movement stops for at least two seconds, we consider another movement [67]
Latency of first contact without aggression (in s)	Seconds that the pig lasts to start the first direct contact with the other pig, which did not follow aggression until five seconds later
Latency of first contact with aggression (in s)	Seconds that the pig lasts to start the first direct contact with the other pig, which followed aggression until five seconds later

Behaviours could occur simultaneously and were not mutually exclusive

A score about aggressiveness was fulfilled. The score 0 was not aggressive: no aggressive contact or aggressive signal/threat, or only one aggressive signal/threat; 1 was aggressive: one aggressive contact and one or more aggressive signals/threats or two or more aggressive signals/threats, and not necessary to stop the encounter; and 2 was very aggressive: more than one aggressive contact and/or more than two aggressive signals/threats or need to stop the encounter due to it (fighting starter).

Besides, the operators noted if the animals eliminated before and/or after the encounter (yes or no – before and/or after) and if it was urine or faeces.

### Biological sample analysis

Just before the assay, saliva samples were thawed and quickly spun to remove putative particulate matter (saliva mucins precipitated due to freezing) that can interfere with the assay. If samples were contaminated by blood (visual inspection), they were discarded. As previously described, the cortisol concentration in saliva was measured using the High sensitivity salivary cortisol enzyme immunoassay kit from Salimetrics® (#1–3002, Salimetrics LLC, State College, USA) following the manufacturer’s instructions. The sensitivity of the assay was 0.007 µg/dL. The mean precision (CV%) obtained in our data was 6.9%. The salivary alpha-amylase activity in the samples was assayed using the Salivary alpha-amylase kinetic enzyme assay kit from Salimetrics® (#1–1902). This kit has been previously validated for use with pig saliva by Fuentes et al. [33]. We followed the manufacturer’s instructions except that we adapted the protocol to pig saliva samples, which were not diluted because of low levels of endogenous alpha-amylase activity. To check that the absence of dilution of the biological matrix did not result in assay interferences, we performed a test of linearity under dilution (serial dilutions from 1:1 to 1:16) on three samples (selected because of their higher alpha-amylase activity) and calculated the coefficient of variation for the corrected concentrations at each dilution [69]. They were all within the acceptable range of 100 ± 20% (mean CV% = 102.4%). In addition, a supplementation assay on undiluted pig saliva samples with the control “High” provided by the manufacturer was also carried out and showed an acceptable recovery (average of 114% for the three samples). These results confirmed the absence of a matrix effect at a dilution of 1:1 (i.e., no dilution) and its suitability for use in this assay kit.

### Statistical analysis

Data analysis was carried out using SAS 9.4 software (Copyright © 2002–2012 by SAS Institute Inc., Cary, NC, USA). The significance threshold was fixed at 5%, which is the standard threshold. The design of the study resulted in two types of data that required different analyses:

1. The data collected per pig correspond to behavioural and physiological data.
2. The data collected per encounter, i.e., the duration of the encounter, the need to stop the encounter and the latencies of first contact with and without aggression.

Before starting the analysis, the inter-observer reliability was evaluated for all the behavioural parameters using the Pearson correlation coefficient, when the assumption of normality was verified, or using the Spearman correlation coefficient, when normality was not verified for at least one of the two observers. This methodology was selected according to Martin and Bateson [70]. The correlations were performed with the CORR procedure, and normality was verified with the UNIVARIATE procedure.

1. Analysis of data collected per pig
  The effects of treatment, sex, day and the treatment × day interaction were initially analysed for behaviour, skin lesions and elimination parameters. For physiological parameters, the effects of treatment, time and the treatment*time interaction were studied. The analyses were performed using mixed models considering the encounter as a random effect. The experimental unit was the pig per encounter. For physiological data, in addition to that, the homogeneity of variances at each sampling time was analysed using Fisher’s tests available in the TTEST procedure.
  For continuous data (behaviours expressed as durations and physiological parameters), general linear mixed models (GLMMs) were carried out. The assumptions of model residual normality and homoscedasticity were checked with QQ plot normality tests and Levene’s test. When conditions were validated, data were transformed using Box‒Cox transformation, and GLMM was performed on transformed data. The GLMMs were computed with the MIXED procedure, normality was verified with the UNIVARIATE procedure, Levene’s test was performed with the GLM procedure, and Box‒Cox transformation was performed with the TRANSREG procedure.
  For discrete data (behaviours expressed as frequency, as well as skin lesions and elimination parameters), mixed models for count data were used. A Poisson mixed model was computed as a first intention. The dispersion was then evaluated by the Pearson chi-square/degrees of freedom (DF) indicator. When overdispersion was detected, the negative binomial mixed model was preferred. These analyses were performed with the GLIMMIX procedure.
  For qualitative data (behaviours expressed as binary measures and scores), mixed logistic regressions were performed for binary parameters, and mixed ordinal logistic regressions were computed for scores with more than 2 classes. These analyses were performed with the GLIMMIX procedure.
  In all these cases, when significant differences were found, multiple comparisons were performed using the Tukey‒Kramer adjustment. In addition, to improve the quality and statistical power of the analyses, the models were simplified step by step as long as the Akaike information corrected criterion (AICc) and Bayesian information criterion (BIC) decreased.
2. Analysis of data collected per encounter
  The effects of treatment and day were studied for the encounter duration and the need to stop the encounter. A 2-way ANOVA was used for the encounter duration for which the assumptions of normality and homoscedasticity were verified. A logistic regression was performed for the need to stop the encounter. Two-way ANOVA and Levene’s test were performed with the GLM procedure, normality was checked with the UNIVARIATE procedure, and logistic regression was computed with the LOGISTIC procedure.
  Latency variables (of the first contact with and without aggression), whose data correspond to delays in the occurrence of an event (in other words, survival data), were analysed. Survival analyses were used to compare treatment groups with the help of the Kaplan‒Meier estimator (using the LIFEREG procedure) and the Cox model (using the LIFEREG procedure).
  The results of the models were accompanied by the descriptive statistics presented as the mean ± standard deviation (SD) for the continuous and discrete data and as frequencies (in percentage) for the qualitative data.

## Results

### Inter-observer reliability

The association between the two observers who performed the video analysis was calculated using Spearman’s correlation coefficient (Table 3). The reliability (inter-observer agreement) was high for all the parameters according to Martin and Bateson [70].

**Table 3:** Inter-observer reliability between the two observers carrying out the video analysis

Parameter	Spearman correlation coefficient (rho)	*P*
Walking	0.98	< 0.0001
Standing inactive	0.98	< 0.0001
Reciprocal look	0.96	< 0.0001
Aggressiveness score	–	
Agonistic behaviour	0.99	< 0.0001
Aggressive signal/threat	0.96	< 0.0001
Fighting	1.00	< 0.0001
Fighting starter	–	
Encounter duration	0.99	< 0.0001
Need to stop the encounter	1.00	< 0.0001
Total social nosing	1.00	< 0.0001
Positive social nosing	0.97	< 0.0001
Negative social nosing	0.95	< 0.0001
Proximity	0.99	< 0.0001
Tail movement	1.00	< 0.0001
Sociability score	–	
Latency of first contact without aggression	0.95	< 0.0001
Latency of first contact with aggression	1.00	< 0.0001

When no correlation coefficient is shown, it is because only one observer analysed the parameter

### Comparisons of the behaviour parameters per mini-pig between the PAP and placebo groups during the encounter

Comparisons of the behavioural parameters of the PAP and placebo groups during the encounter were performed using the video recordings and the video analysis of the two independent observers.

Concerning fighting (binary), a significant difference was found between the PAP and placebo groups (df = 1; Fisher statistic = 13.47; *P* = 0.001; Mixed Logistic Regression), being lower in the PAP group than in the placebo group (36.36% vs. 63.64%, respectively).

Several trends were found between the PAP and placebo groups. Regarding aggressiveness score, the mini-pigs of the PAP group showed a trend to be scored as 0 (not aggressive at all), while the mini-pigs of the placebo group showed a trend to be scored as 2 (very aggressive) (df = 1; Fisher statistic = 3.61; *P* = 0.07; Mixed Ordinal Logistic Regression). For agonistic behaviour (binary), the mini-pigs of the PAP group attacked 1.9 times less the other mini-pig than mini-pigs of the placebo group (34.78% vs. 65.22%) (df = 1; Fisher statistic = 4.14; *P* = 0.058; Mixed Logistic Regression). Regarding immobile and/or ground sniffing (duration in seconds), the time spent by the mini-pigs immobile and/or ground sniffing was two times higher for the PAP group than that of the placebo group (150.85 ± 115.44 s vs. 70.87 ± 96.05 s, respectively) (df = 1; Fisher statistic = 3.18; *P* = 0.088; General Linear Mixed Model). Concerning reciprocal look (duration in seconds), the mini-pigs of the PAP group tended to spend more time looking at each other reciprocally than the mini-pigs of the placebo group (23.38 ± 29.80 s vs. 12.46 s ± 20.90 s, respectively) (df = 1; Fisher statistic = 3.59; *P* = 0.071; General Linear Mixed Model). Finally, a trend was also found for positive social nosing (binary), as the mini-pigs of the PAP group tended to show less positive social nosing than the mini-pigs of the placebo group (37.50% vs. 62.50%) (df = 1; Fisher statistic = 4.24; *P* = 0.055; Mixed Logistic Regression).

No significant differences were found in terms of walking, agonistic signal/threat, starting fights, proximity, tail movement or negative social nosing (Table 4).

**Table 4:** Descriptive data of nonsignificant behavioural parameters per pig

Parameters	Statistical indicator	PAP	Placebo	Treatment		
				DF	*F*	*P*
Walking (duration in sec)	Mean ± SD	29.05 ± 21.61	36.10 ± 32.18	1.00	0.40	0.53
Aggressive signal/threat (frequency)	Mean ± SD	1.35 ± 1.18	2.42 ± 2.83	1.00	1.68	0.21
Fighting starter (binary)	Frequency in % No (0) Yes (1)	52.78% 41.67%	47.22% 58.33%	1.00	0.69	0.42
Negative social noising (binary)	Frequency in % No (0) Yes (1)	50.00% 50.00%	50.00% 50.00%	1.00	0.00	0.97
Proximity (duration in s)	Mean ± SD	75.90 ± 59.21	72.60 ± 76.28	1.00	0.05	0.83
Tail movement (duration in s)	Mean ± SD	50.30 ± 77.02	40.51 ± 60.25	1.00	0.00	0.98

Statistical analysis: Simplified GLMM for the durations (walking, proximity, tail movement), mixed Poisson model for aggressive signal/threat, mixed logistic regression for fighting starter and mixed ordinal logistic regression for the sociability score

### Comparisons of pair behaviour parameters between the PAP and placebo groups during the encounter

Concerning the latency of the first contact without aggression (in seconds) (survival analysis), a significant treatment effect was found between the PAP and placebo groups on the latency of the first contact without aggression (df = 1; χ^2^ = 4.74; *P* = 0.0295; Cox Model). Mini-pigs receiving PAP made the first contact without aggression at an average of 4.33 ± 4.84 s (mean ± SD), while those receiving placebo made the first contact without aggression at an average of 78.08 ± 95.07 s.

For the encounter duration, the need to stop the encounter and the latency of the first contact with aggression, no significant differences were found (Table 5).

**Table 5:** Descriptive data of nonsignificant behavioural parameters per pair

Parameter	Statistical indicator	PAP	Placebo	Statistical analysis	Treatment		
					DF	Statistic	*P*
Encounter duration (in s)	Mean ± SD	205.29 ± 116.19	152.30 ± 129.39	Two-way ANOVA (reduced model)	1.00	1.10	0.31
Need to stop the encounter (binary)	Frequency in % No (0)	60.00%	40.00%	Simplified logistic regression	1.00	1.20	0.27
	Yes (1)	38.46%	61.54%				
Latency of first contact with aggression (in s)	Mean ± SD	67.30 ± 75.06	43.89 ± 52.06	Cox model	1.00	1.59	0.21

### Comparison of salivary cortisol concentration (µg/dL) between the PAP and placebo groups

**Fig. 2:**
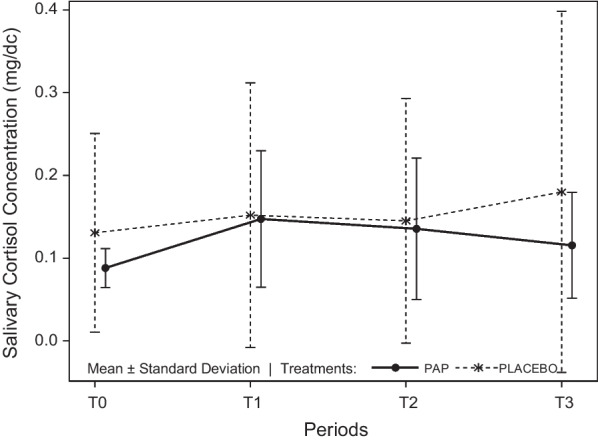
Mean salivary cortisol concentration (µg/dL) ± Standard Deviation per group and time. T_0_: forty minutes before the encounter; T_1_: just after the end of the encounter; T_2_: fifteen minutes after the end of the encounter; T_3_: thirty minutes after the end of the encounter. No significant differences were observed

No significant difference was found between treatments (df = 1; Fisher statistic = 0.10; *P* = 0.752; general linear mixed model) or between times (df = 3; Fisher statistic = 1.96; *P* = 0.123) (Table 6 and Fig. 2).

**Table 6:** Descriptive data of salivary cortisol (µg/dL) by treatment and time

Treatment A (PAP)						Treatment B (Placebo)					
Time	N	Mean	Minimum	Maximum	Std Dev	Time	N	Mean	Minimum	Maximum	Std Dev
T0	24	0.09	0.05	0.17	0.02	T0	24	0.13	0.05	0.64	0.12
T1	22	0.15	0.05	0.32	0.08	T1	24	0.15	0.06	0.69	0.16
T2	23	0.14	0.06	0.45	0.09	T2	24	0.15	0.04	0.75	0.15
T3	23	0.12	0.04	0.36	0.06	T3	23	0.18	0.05	0.83	0.22

Additionally, the homogeneity of variances between the PAP and placebo groups was analysed at each time point. At T_0_, variances were heterogeneous (SD PAP: 0.02 vs. SD placebo: 0.12; df = 23; Fisher statistic = 26.00; *P* < 0.001; Fisher’s test). At T_1_, they were also heterogeneous (df = 23, Fisher statistic = 3.75; *P* = 0.003; Fisher’s test). At T_2_, homogeneity was also not verified (df = 23; Fisher statistic = 3.00; *P* = 0.012; Fisher’s test), nor at T_3_ (df = 22; Fisher statistic = 11.63; *P* < 0.001; Fisher’s test) (Table 6). This means that the variability over time within each group was different, being less dispersed in the PAP group.

### Comparison of salivary alpha-amylase activity (U/mL) between the PAP and placebo groups

**Fig. 3:**
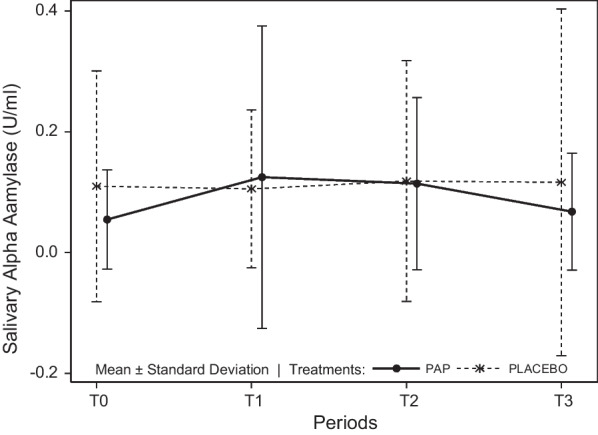
Mean salivary alpha-amylase activity (U/mL) ± Standard Deviation per group and time. T_0_: forty minutes before the encounter; T_1_: just after the end of the encounter; T_2_: fifteen minutes after the end of the encounter; T_3_: thirty minutes after the end of the encounter. No significant differences were observed

No significant difference was found between treatments (df = 1; Fisher statistic = 0.25; *P* = 0.621; general linear mixed model) or between times (df = 3; Fisher statistic = 1.65; *P* = 0.1874) (Table 7 and Fig. 3).

**Table 7:** Descriptive data of salivary alpha-amylase activity (U/mL) by treatment and time

Treatment A (PAP)						Treatment B (Placebo)					
Time	N	Mean	Minimum	Maximum	Std Dev	Time	N	Mean	Minimum	Maximum	Std Dev
T0	23	0.05	0.00	0.34	0.08	T0	20	0.11	0.01	0.83	0.19
T1	22	0.12	0.00	0.94	0.25	T1	20	0.11	0.00	0.45	0.13
T2	22	0.11	0.00	0.57	0.14	T2	20	0.12	0.00	0.82	0.20
T3	23	0.07	0.00	0.45	0.10	T3	20	0.12	0.01	1.30	0.29

Concerning the homogeneity of variances at each time, variances were compared at T_0_ for both groups, showing that they were heterogeneous (SD PAP: 0.08 versus SD placebo: 0.19; df = 22; Fisher statistic = 5.39; *P* < 0.001; Fisher’s test). At T_1_, they were heterogeneous (df = 19, Fisher statistic = 3.67; *P* = 0.006; Fisher’s test), meaning that the variability inside each group was different, being less dispersed in the PAP group. At T_2_, homogeneity of variances between PAP and placebo groups was verified (df = 19; Fisher statistic = 1.96; *P* = 0.130; Fisher’s test). Finally, at T_3_, variances were heterogeneous (df = 19; Fisher statistic = 8.81; *P* < 0.001; Fisher’s test) (Table 7).

### Comparison of skin lesions and elimination between the PAP and placebo groups

Concerning skin lesions (PAP 0.625 ± 1.55 vs. placebo = 0.292 ± 0.75; df = 1; Fisher statistic = 0.90; *P* = 0.353; Poisson mixed model) and elimination (PAP = 0.75 ± SD: 0.99 vs. placebo = 0.54 ± 0.59; df = 1; Fisher statistic = 0.16; *P* = 0.691; Poisson mixed model), no significant difference between treatments was found.

## Discussion

The aim of the present study was to investigate the effect of the new application of pig appeasing pheromone applied on withers skin as a model of pig mixing to determine whether (i) it reduces aggression and fighting and (ii) it impacts behavioural and physiological indicators of positive and negative welfare.

Concerning behavioural parameters, the results showed that the PAP applied on withers skin reduces fighting between pigs. In addition, according to some trends, the results suggested that the pigs were less aggressive, as they were more often scored as not aggressive at all and attacked the other mini-pigs less than when placebo was applied.

Interestingly, the results suggested that when PAP was applied, the mini-pigs tended to spend more time looking at each other reciprocally than when placebo was applied, which could be a sort of prosocial behaviour [9, 21, 22], even if further research would be needed to confirm it, as those behaviours in pigs are still relatively unclear [18, 22]. Finally, the mini-pigs with the PAP tended to spend more time immobile and/or ground sniffing than the mini-pigs with the placebo; thus, they were calmer, which could be an indicator of animal welfare [71].

One limitation of the study was the low sample size, being important thus to confirm the results with a higher number of animals in real conditions with domestic commercial pigs. In that case, the study of the role of individual differences could be analysed. Another limitation was the inaccuracy of differentiating positive and negative social nosing. In fact, total social nosing was measured and then divided into positive or negative. Positive social nosing was considered when no aggression or fighting appeared after five seconds, and negative social nosing when it appeared. Nevertheless, it is difficult to know if, when no aggression appeared after social nosing, this absence was due to this sort of positive or neutral contact or because of a sort of threat from one animal to the other, which would be considered negative contact (and would thus avoid the final aggression or fighting). Therefore, this inaccuracy of the parameter could explain the result, which was that the pigs of the PAP group showed less “positive” social nosing than the pigs of the placebo group. In fact, Camerlink et al. [9] showed that social nosing towards unfamiliar conspecifics might be more likely related to recognition and becoming acquainted with each other (more than an affiliative behaviour), which may include sorting out dominance relationships. The same happened with the latency of the first contact with aggression and without aggression. The first could be considered negative contact because following the first contact, aggression appeared. However, the second could be either considered a positive contact (a sort of prosocial behaviour), which would avoid the appearance of the aggression, or a negative contact (a sort of threat), which also avoided the appearance of the aggression. Thus, concerning the parameter first contact without aggression, it is difficult to interpret the result because there are many different possibilities. One possible hypothesis is that this first contact might be a threat; therefore, there might be fewer threats with PAP than with placebo, but further research is needed to confirm this hypothesis.

Regarding the physiological parameters, and more precisely, salivary cortisol concentrations, no significant difference was found. The homogeneity of variances between the PAP and placebo groups was not verified at any time (T_0_, T_1_, T_2_ and T_3_), which means that they were heterogeneous. At every time point, the variability (dispersion) within each group was lower when PAP was applied than when placebo was applied. This variability dramatically increased at T_3_, where we observed that there was much more variability in the placebo group than in the PAP group. Interestingly, T_3_ (30 min after the event) was when the effects of the encounter on cortisol should be observed in saliva according to its kinetics of passive diffusion from blood to saliva [33]. This result implied that, with PAP application, there was a small intra-group difference regarding the cortisol concentrations, but there was a large intra-group difference in the placebo group, and this difference increased at T_3_. This suggested that the cortisol levels of the mini-pigs receiving PAP were more homogenous and more stable within the group, and these animals may better cope with a stressful situation (encounter with an unfamiliar conspecific) than mini-pigs in the placebo group, in which individuals displayed very variable cortisol concentrations, including very high ones, reflecting very different stress responses, including very strong ones (see maximum values in Table 5). Given the homogenisation of the cortisol stress responses in the PAP group, it seems that the maternal appeasing pheromone can balance/smooth the stress responses mediated by the LHPA axis in animals, suggesting that animals receiving the maternal appeasing pheromone could better cope with stressful situations that the mini-pigs without it, by preventing some animals from reacting very strongly and detrimentally to these events. This means that these results suggest a more stable emotional balance or better resilience to stressful events thanks to pheromones. In addition, the graphical representation of the salivary cortisol concentrations over time (Fig. 2) showed that, even if not significantly, at T_3_, the mean cortisol concentration decreased in the PAP group but increased in the placebo group. Finally, it is possible that the vast dispersion of cortisol concentrations at T_3_ in the placebo group could prevent reaching the statistically significant difference from the PAP group.

Concerning salivary alpha-amylase activity, no significant difference was found. Homogeneity of variances between the PAP and placebo groups was not verified at T_0_, T_1_ or T_3_ and was thus heterogeneous, except at T_2_. These results suggested that, even if at T_1_, the mini-pigs of the PAP group displayed a more variable alpha-amylase activity, while at T_2_, this variability (dispersion) decreased. Then, at T_3_, there was less variability in the PAP group. Interestingly, the effect of the encounter on the alpha-amylase activity was supposed to be similar between T_2_ and T_3_ (20–30 min after the stressful event), according to the literature [33]. Therefore, the effects of the PAP were seen at that time, suggesting that the animals were more stable, more balanced, and coped better with the stress thanks to the pheromone, while with placebo, at T_3_, the individuals showed very different levels of alpha-amylase activity. In addition, the graphical representation of the mean salivary alpha-amylase activity over time (Fig. 3) showed that, even if not significant, the alpha-amylase activity was lower with the pheromone than with placebo at T_3_. These results confirmed that in the presence of PAP, pig alpha-amylase activity was more balanced/homogenous, as was the cortisol level, which are both indicators of stress and thus poor welfare. Therefore, this suggests that PAP helped the animals cope with a stressful situation and homogenised the stress response (or absence of stress response) within a group.

Concerning skin lesions and elimination, no significant differences were found. In future studies, more (long-term) parameters will be included, which was not possible in this study, as the PAP, even if with another mode of application, had already shown improvements in weight gain and feeding stimulation in weaned pigs [41] and in feed intake in weaners [38].

The present study thus suggests that PAP applied on withers skin (i) reduces fighting and aggression in mini-pigs; (ii) seems to increase some prosocial behaviours (e.g. reciprocal look), although further research is needed for this group of parameters; and (iii) improve behavioural and physiological indicators of animal welfare. Although several farmers in the pig industry have already expressed in different ways their contentment with the product SecurePig Flash® (thus, the new application of SecurePig) and the described effects on their own commercial pigs, to our knowledge, this is the first scientific study demonstrating them. Nevertheless, further research would be needed. Thus, the next step would be to investigate all these parameters, as well as others, with domestic commercial pigs under farm conditions.

## Conclusions

SecurePig Flash® (pig appeasing pheromone) seems to help improving the welfare of pigs by reducing fighting, aggression and stress, among other positive effects already shown in previous studies about another mode of application of the pheromone. It seems thus an interesting biomimetic tool to use in pig production systems to improve their welfare in a feasible way.

## Data Availability

The datasets generated or analysed during this study are available from the corresponding author on reasonable request.

## Abbreviation

PAP: Pig appeasing pheromone
